# 3D profiling of mouse epiphyses across ages reveals new potential imaging biomarkers of early spontaneous osteoarthritis

**DOI:** 10.1111/joa.13834

**Published:** 2023-02-11

**Authors:** Eva C. Herbst, Lucinda A. E. Evans, Alessandro A. Felder, Behzad Javaheri, Andrew A. Pitsillides

**Affiliations:** ^1^ Palaeontological Institute & Museum University of Zurich Zurich Switzerland; ^2^ Skeletal Biology Group, Comparative Biomedical Sciences Royal Veterinary College London UK; ^3^ Research Software Development Group, Advanced Research Computing University College London London UK

**Keywords:** biomarkers, cortical bone, epiphysis, imaging, micro‐CT, mouse model, osteoarthritis, trabecular bone

## Abstract

Worldwide research groups and funding bodies have highlighted the need for imaging biomarkers to predict osteoarthritis (OA) progression and treatment effectiveness. Changes in trabecular architecture, which can be detected with non‐destructive high‐resolution CT imaging, may reveal OA progression before apparent articular surface damage. Here, we analysed the tibial epiphyses of STR/Ort (OA‐prone) and CBA (healthy, parental control) mice at different ages to characterise the effects of mouse age and strain on multiple bony parameters. We isolated epiphyseal components using a semi‐automated method, and measured the total epiphyseal volume; cortical bone, trabecular bone and marrow space volumes; mean trabecular and cortical bone thicknesses; trabecular volume relative to cortical volume; trabecular volume relative to epiphyseal interior (trabecular BV/TV); and the trabecular degree of anisotropy. Using two‐way ANOVA (significance level ≤0.05), we confirmed that all of these parameters change significantly with age, and that the two strains were significantly different in cortical and trabecular bone volumes, and trabecular degree of anisotropy. STR/Ort mice had higher cortical and trabecular volumes and a lower degree of anisotropy. As the two mouse strains reflect markedly divergent OA predispositions, these parameters have potential as bioimaging markers to monitor OA susceptibility and progression. Additionally, significant age/strain interaction effects were identified for total epiphyseal volume, marrow space volume and trabecular BV/TV. These interactions confirm that the two mouse strains have different epiphyseal growth patterns throughout life, some of which emerge prior to OA onset. Our findings not only propose valuable imaging biomarkers of OA, but also provide insight into ageing 3D epiphyseal architecture bone profiles and skeletal biology underlying the onset and development of age‐related OA in STR/Ort mice.

## Graphical abstract

We analysed the tibial epiphyses of osteo‐arthritis prone (STR/Ort, blue in image below) and healthy (CBA, red in image below) mice to characterise the effects of mouse age and strain on multiple bony parameters. The two strains were significantly different in cortical and trabecular bone volumes, and trabecular degree of anisotropy; these parameters have potential as bioimaging markers to monitor OA susceptibility and progression. Additionally, the two mouse strains have different epiphyseal growth patterns throughout life, as revealed by significant age/strain interaction effects for total epiphyseal volume, marrow space volume and trabecular BV/TV.

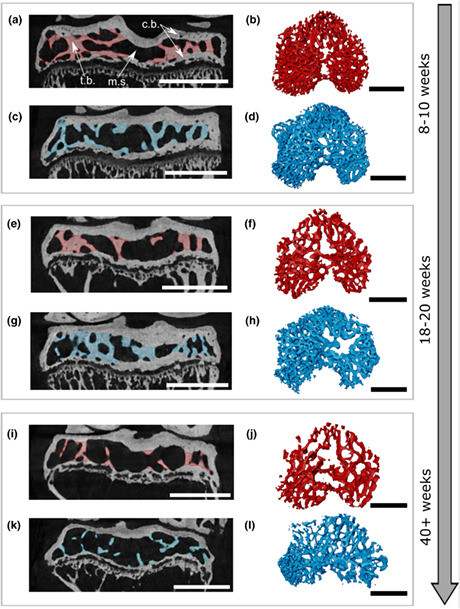

## 1 INTRODUCTION

Osteoarthritis (OA) is a widespread and painful process of joint degeneration that impacts millions of people worldwide (Ghouri & Conaghan, 2021). Despite its high prevalence, there is a dearth of standardised clinical methods by which to evaluate OA predisposition, progression and treatment efficacy. Identification of structural changes in early OA is a useful avenue of research because of the potential for early diagnosis and validation for OA treatment. Indeed, focus groups all over the world agree that new biomarkers to phenotype and predict the progression of OA are a worthy research priority (Conaghan et al., 2014; Ghouri & Conaghan, 2021; Hunter et al., 2019).

Trabecular structure is emerging as a promising predictive imaging biomarker of OA; changes in architecture have been correlated to OA progression and these modifications can be evaluated in OA patients. Trabecular bone volume fraction and thickness were greater in OA than in healthy humans in medial tibial condyle cross‐sections (Kamibayashi et al., 1995). Trabecular bone/total volume (BV/TV) and number increased in superior human femur sections, whereas thickness decreased in the inferomedial femur in OA (Fazzalari & Parkinson, 1997). Contrastingly, Patel et al. (2003) found lower trabecular BV/TV and thickness in small cores of the proximal human tibial plateau in OA. Such changes may have functional impact as trabecular bone is believed to serve as an impact‐dampener within load‐bearing joints (Seeman, 2008), and it is also responsive to local stresses (Reznikov et al., 2020), as evidenced by the differing medial and lateral trabecular organisation in valgus versus varus OA and non‐OA subjects (Rapagna et al., 2021; Renault et al., 2020; Roberts et al., 2017).

Fractal texture analysis has also been used to explore links between trabecular anatomy and OA pathobiology. Fractal texture analysis provides a fractal dimension measurement, which can be taken as a measure of trabecular *roughness* (Janvier et al., 2017). Fractal dimensions were found to differ between patients with progressive OA (vs non‐progressive OA), and between non‐OA and severe OA, (Fazzalari & Parkinson, 1997; Janvier et al., 2017; Kraus et al., 2018). These data suggest that the tracking of trabecular organisation may provide an early and predictive imaging biomarker for OA. Recent advances in CT scanning resolution enable complete 3D analyses of trabecular structures and it is, therefore, an opportune time to investigate the bony changes associated with OA.

As with any bony changes, the question of whether atypical trabecular anatomy is a *cause* or *effect* of OA will always be challenging to address in human‐centric research, as a pre‐OA state in vulnerable individuals cannot be readily identified from scans of healthy joints. Although this may change as in vivo clinical imaging methods continue to evolve and novel imaging biomarkers are developed, bony changes nonetheless currently remain the earliest observable symptoms of human OA onset (He et al., 2014; Kraus et al., 2018). In this respect, inbred animal models with a predictable age of OA onset provide a clear advantage for the detection of a pre‐OA state.

The value of trabecular analyses will likely be enhanced by correspondent evaluation of other anatomical joint structures, as OA is a *whole‐joint* disease in which no single part appears to be affected independently (Guermazi et al., 2013). Finnila et al. (2017) found that the subchondral bone plate (SCBP) thickened with increasing OA severity in human tibias. Das Neves Borges et al. (2017) found an increase in SCBP thickness and medial osteophyte volume in the tibia of mice with OA induced by destabilisation of medial meniscus. This cortical bone expansion occurred with an apparent decrease in the size of the trabecular compartment. These data highlight the value of evaluating complex spatial interrelationships across multiple compartments and that analyses aiming to identify imaging biomarkers in the bone should extend to the corticalised compartments, including the SCBP.

Subchondral bone evaluation typically involves selection of cubic or cylindrical core volumes of interest (VOIs) to measure 3D parameters (Cox et al., 2012; Finnila et al., 2017; Florea et al., 2015; Renault et al., 2020). This enables the rapid sampling without requisite segmentation of trabecular bone from the cortex and marrow space. Unlike VOIs, whole‐structure segmentations (e.g. entire epiphysis) allow 3D analysis of all relevant trabecular and cortical bone, reducing 2D edge‐artefacts where VOIs are artificially ‘cut off.’ Whole‐structure segmentation may indeed be most useful in the study of OA, where the entire joint anatomy may be disrupted. For example, extensive *lipping* and broadening of the lateral part of a condyle in an OA joint may mean that a VOI numerically calculated as the *centre* of that condyle would, in practice, be anatomically more laterally located than the identically calculated *centre* of the matched condyle in a healthy joint. This could yield false positive differences detected between healthy and OA bone architectures in allegedly location‐matched VOIs. Measures of complex anatomy, such as trabecular anisotropy, are also unlikely to yield meaningful data if a joint sub‐region containing few trabeculae is selected. Moreover, small VOIs reduce trabecular sample number, thus increasing the risk of an anomalous region being inaccurately pinpointed as representative of the entire joint. 3D analyses of entire joints are thus desirable in the exploration of these vital structural relationships. New scanners that can image human trabeculae in vivo make these architectural imaging biomarkers clinically accessible, and yet the specific 3D parameters that can be used to predict and monitor the progression of OA remain to be identified.

The value of such studies is likely bolstered by the examination of bone changes in animal models. In a rabbit injury‐induced femur OA model, SCBP and trabecular BV/TV were markedly decreased in the medial condyle, likely due to reduced loading, whilst cartilage changes were pronounced laterally (Florea et al., 2015). As most human OA cases are age‐ rather than injury‐induced (Shane Anderson & Loeser, 2010), there is likely added value in probing these parameters in animal models such as the STR/Ort mouse that spontaneously develops progressive OA from 16 to 20 weeks of age.

We developed an open‐access, semi‐automated method enabling segmentation of the tibial epiphysis from μCT scans, which can then isolate cortical from trabecular bone and marrow space as non‐overlapping 3D volumes for quantitative analysis (Herbst et al., 2021). Here, we apply our method to analyse both the entire STR/Ort epiphyses and the closely related parental CBA control strain, to identify reliable imaging biomarkers for OA. We hypothesise that quantifiable epiphyseal differences are present between the two strains at 8–11 weeks of age before OA onset, which may partly underlie future predisposition to serve as ‘pre‐OA’ biomarkers translatable into human clinical practice. Likewise, we anticipate clinically useful epiphyseal differences to be detectable between the strains at 18–20 (early‐OA) and 40+ weeks (late‐stage OA) of age. We concentrate on the tibial epiphysis as this is first affected by OA in the STR/Ort strain (Staines et al., 2017). We include both the medial and lateral compartments of tibial epiphyses in our analysis because fractal analysis of trabecular architecture demonstrated that including both compartments resulted in the best predictive model for OA (Janvier et al., 2017).

Our work follows on from the elegant studies of Stok et al. (2009), who used μCT to compare 3D bone structure at different locations and disease time points in STR/Ort with CBA mice. Stok et al. differentiated epiphyseal cortical from trabecular bone, as we have done, but excluded the epiphyseal layer of solid bone adjacent to the growth plate (distal to epiphyseal trabeculae); our method includes this as a component of the epiphyseal cortex. Our research examined both shared and different parameters, allowing us to both extend and confirm the reproducibility of their findings. Our study additionally and uniquely reports upon trabecular anisotropy; marrow space volume; trabecular volume relative to cortical volume, and total epiphyseal volume. Our focus is on highlighting imaging biomarkers of early‐ or pre‐OA in younger mice (8–11 weeks) in this uniquely OA‐prone strain.

## 2 METHODS

We compared tibial epiphyses of male OA (STR/Ort) and healthy control (CBA) mice in three age groups: 8–11 weeks (a pre‐OA timepoint of skeletal immaturity), 18–20 weeks (representing greater skeletal maturity, and the earliest‐detected OA timepoint in STR/Ort mice) and 40+ weeks (representing ageing, and late‐stage OA for the STR/Ort mice). Thus, mouse strain represents OA predisposition, and age represents OA progression. All mice were killed using cervical dislocation or rising CO_2_, either as part of previous RVC research studies (Javaheri et al., 2018) or mouse colony control. Hindlimbs were removed, fixed in 4% PFA for 24–48 h, and stored long‐term in the Royal Veterinary College sample archive following serial transfer into 70% ethanol. Archive samples were used for ethical reasons to reduce the number of mice bred and killed for research purposes. Unfortunately, historic sample labelling did not entirely conform to current ARRIVE guidelines, meaning that we are unable to provide individual mouse body weight information or exact age (in days). However, both strains share a documented tendency towards obesity, and age groups of all mice are known. Five individuals were included for all groups. Samples were scanned using desktop X‐ray micro‐computed tomography (micro‐CT, Bruker Skyscan 1172, isometric voxel size 5 μm, exposure time 1600 ms and 0.06 degree rotation angle).

We implemented the semi‐automated segmentation algorithm developed by Herbst et al. (2021) to isolate each entire tibial epiphysis and segment from them the trabecular bone, cortical bone (including articular calcified cartilage), and marrow space into three separate non‐overlapping 3D volumes in Avizo. We sealed large foramina manually for a high‐precision segmentation, although sensitivity studies in mouse epiphyses show that foramen‐sealing does not greatly affect parameters such as trabecular BV/TV, trabecular volume/cortical volume and trabecular degree of anisotropy (Herbst et al., 2021). Isolated epiphyses were viewed as 2D image stacks and as a 3D model, at which point four damaged specimens were identified and discarded. Final sample sizes are reported in Table 1. Entire epiphyses, including both condyles and the intercondylar region, were analysed. Growth plate bridges between the epiphysis and metaphysis were excluded from all analyses. Avizo (v 2019.2, Thermo Fisher Scientific) was used to measure the following parameters: degree of trabecular anisotropy (values on a scale from 0: fully isotropic, to 1: fully anisotropic), trabecular volume (mm^3^), cortical volume (mm^3^), total epiphyseal volume (mm^3^), marrow space volume (MSV, mm^3^) and trabecular BV/TV (calculated as: trabecular volume/ (marrow space volume + trabecular volume)). Trabecular and cortical thicknesses (mm) were calculated in Fiji using the thickness plugin in BoneJ2 version 1.11.2 (Domander et al., 2021; Doube et al., 2021; Rueden et al., 2017).

**TABLE 1:** Number, strain and ages of the male mouse epiphyses included in this study.

Strain	Model	Age (weeks)		
		8–11	18–20	40+
CBA	Healthy control	5	5	5
STR/Ort	Osteoarthritic	5	5	5

All parameters were subject to Levene's test in SPSS, with a null hypothesis of homogenous distribution of error variances. A two‐way multivariate ANOVA in SPSS was used to analyse all parameters with non‐significant outcomes, with the two fixed factors defined as mouse strain (either STR/Ort or CBA) and age group (either 8–11 weeks, 18–20 weeks or 40+ weeks). *p*‐values ≤0.05 were deemed significant throughout. Where age groups differed significantly in a given parameter, a Tukey's post hoc test was applied. In some cases, significant interaction effects between mouse strain and age were also identified. These interactions indicate that in some epiphyseal parameters, the two strains of mice differ from one another specifically regarding the way in which they age. Post hoc full simple main effects analysis using Fisher's least significant differences (LSD) was then performed to clarify these relationships. For those parameters in which significantly different error variances were detected by initial Levene's test, a non‐parametric independent‐samples Kruskal–Wallis test with post hoc pairwise comparisons was performed instead of ANOVA, to identify any statistical differences between the groups shown in Table 1.

## 3 RESULTS

### 3.1 Ageing affects all epiphyseal parameters, but is only the exclusive determinant of trabecular‐to‐cortical volume ratio

**FIGURE 1:**
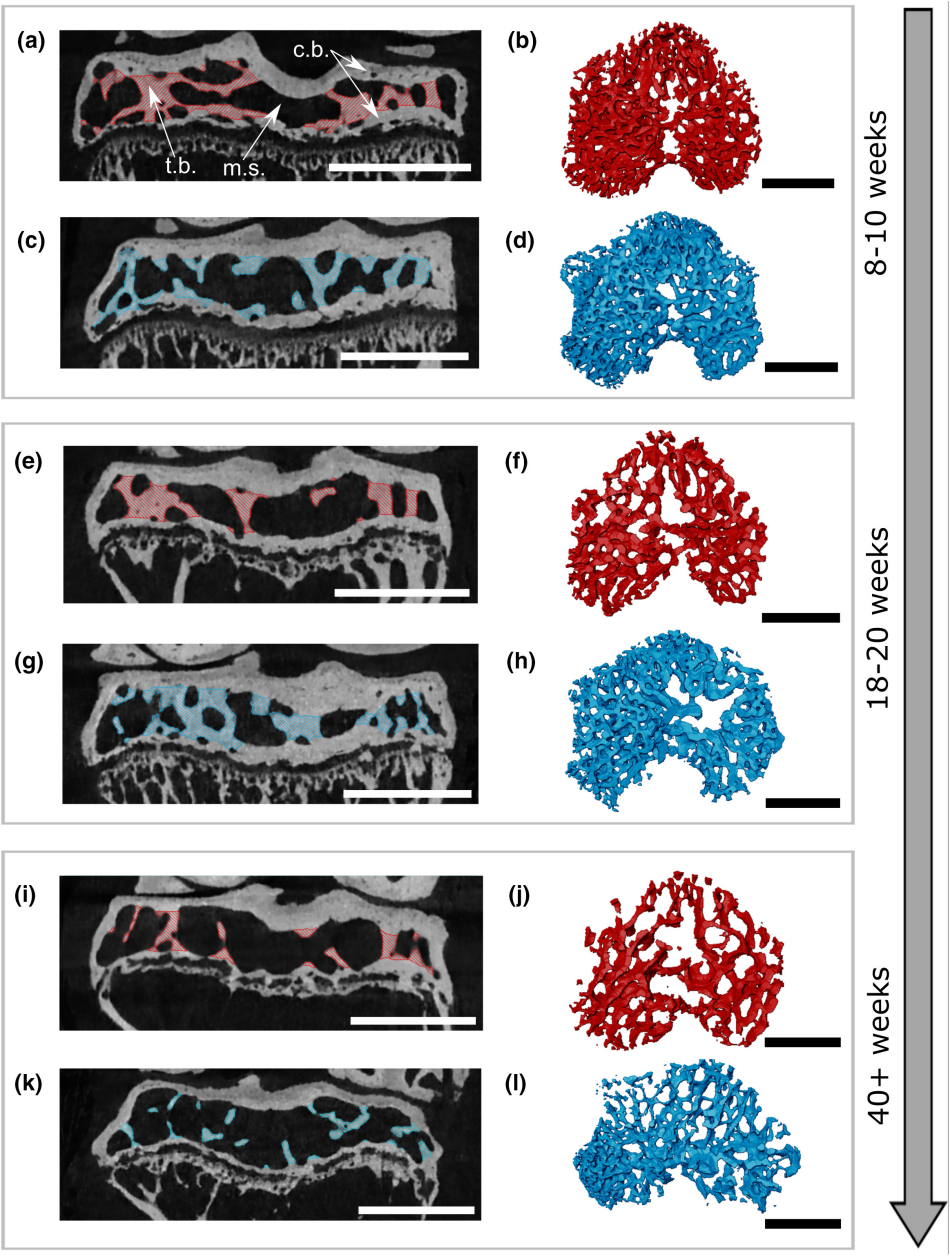
Trabecular segmentations of mouse tibia epiphyses. Red, CBA (healthy) and blue, STR/Ort (OA) trabeculae. Left‐hand side images (a, c, e, g, i, k) show central slices from reconstructed μCT scans viewed in coronal plane, with segmented trabeculae highlighted (blue or red). Right‐hand side images (b, d, f, h, j, l) show corresponding trabeculae rendered as 3D volume, viewed from above. (a/b) 8–11 week CBA; (c/d) 8–11 week STR/Ort; (e/f) 18–20 week CBA; (g/h) 18–20 week STR/Ort; (i/j) 40+ week CBA; (k/l) 40+ week STR/Ort. Age groups are indicated on right‐hand side. Scale bars = 1 mm. c.b., cortical bone, m.s., marrow space; t.b., trabecular bone.

Following confirmation by eye that semi‐automated segmentations of epiphyses and their components were successful (Figure 1), two‐way ANOVA and Kruskal–Wallis tests were applied to reveal that all bone parameters showed statistically significant changes with age (Tables 2, 3). We observed that standard deviations for parameters measured in STR/Ort mice were frequently greater than those for CBA counterparts, especially in the older age group (Table 4).

**TABLE 2:** *p*‐values identified from two‐way ANOVA and Levene's tests.

	Strain	Age	Strain × age interaction	Levene's test
Total epiphyseal volume	0.004*	<0.001*	0.012*	0.267
Cortical bone volume	<0.001*	<0.001*	0.963	0.277
Trabecular bone volume (mm^3^)	0.001*	<0.001*	0.261	0.355
Marrow space volume (MSV)	0.129	<0.001*	<0.001*	0.447
Trabecular BV/TV	<0.001*	<0.001*	0.005*	0.301
Trabecular volume: cortical volume	0.369	<0.001*	0.628	0.211
Trabecular degree of anisotropy	0.002*	0.017*	0.382	0.554
Trabecular thickness (mm)	n/a	n/a	n/a	0.029*
Cortical thickness (mm)	0.040*	<0.001*	0.473	0.106

*Note*: Levene's test was operated with a null hypothesis of homogenous distribution of error variances, based on median values with adjusted degrees of freedom.

* Indicates significance at the 0.05 level.

**TABLE 3:** *p*‐values identified from non‐parametric independent‐sample Kruskal–Wallis test of mean trabecular thickness (mm).

Pairwise comparison of mean trabecular thickness (mm), between:	*p*‐value
8–11 week CBA–18–20 week CBA	0.006*
8–11 week CBA–40 + week CBA	0.179
18–20 week CBA–40 + week CBA	0.315
8–11 week STR–18–20 week STR	0.429
8–11 week STR–40 + week STR	0.172
18–20 week STR–40 + week STR	0.565
8–11 week CBA–8–11 week STR	0.161
18–20 week CBA–18–20 week STR	0.184
40 + week CBA–40 + week STR	0.801

* Indicates significance at the 0.05 level.

**TABLE 4:** Mean of all epiphyseal bone parameters measured. Standard deviation is reported in parentheses.

	8–11 weeks		18–20 weeks		40+ weeks	
	CBA	STR/ort	CBA	STR/ort	CBA	STR/ort
Total epiphyseal volume (mm^3^)	2.805 (0.164)	2.829 (0.332)	2.698 (0.186)	3.242 (0.195)	3.182 (0.051)	3.283 (0.105)
Cortical bone volume (mm^3^)	1.143 (0.054)	1.350 (0.208)	1.520 (0.107)	1.760 (0.107)	1.570 (0.062)	1.780 (0.245)
Trabecular bone volume (mm^3^)	0.407 (0.072)	0.514 (0.059)	0.337 (0.048)	0.421 (0.034)	0.305 (0.026)	0.319 (0.052)
Marrow space volume (MSV, mm^3^)	1.253 (0.118)	0.966 (0.090)	0.841 (0.105)	1.058 (0.118)	1.304 (0.037)	1.235 (0.153)
Trabecular BV/TV	0.245 (0.037)	0.347 (0.022)	0.286 (0.012)	0.286 (0.020)	0.190 (0.016)	0.207 (0.044)
Trabecular volume relative to cortical volume	0.357 (0.065)	0.383 (0.022)	0.222 (0.033)	0.239 (0.025)	0.195 (0.017)	0.189 (0.043)
Trabecular degree of anisotropy	0.517 (0.007)	0.513 (0.008)	0.529 (0.004)	0.516 (0.003)	0.518 (0.006)	0.507 (0.011)
Trabecular thickness (mm)	0.056 (0.002)	0.061 (0.003)	0.067 (0.002)	0.063 (0.002)	0.064 (0.002)	0.066 (0.008)
Cortical thickness (mm)	0.086 (0.006)	0.098 (0.011)	0.126 (0.009)	0.127 (0.008)	0.125 (0.002)	0.136 (0.019)
Total standard deviation from all parameters	0.525	0.733	0.506	0.512	0.219	0.680

**FIGURE 2:**
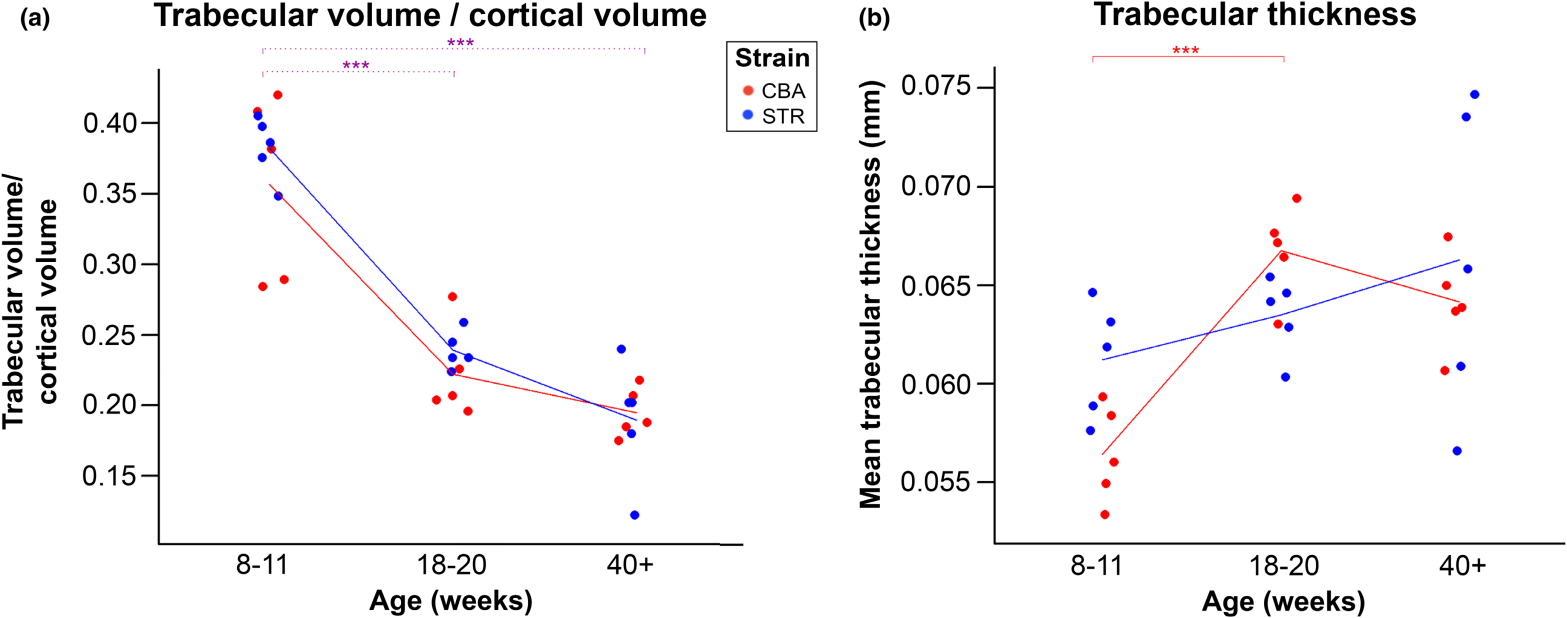
Parameters affected by mouse age but with no significant difference between strains at any of the time points. (a) Epiphyseal trabecular volume relative to cortical volume and (b) trabecular thickness was significantly affected by mouse age in younger CBA mice, but with no significant difference identified between the two strains at any given age. Lines on plot connect mean values distinguishing between STR/Ort (OA, blue) and CBA (healthy, red) mice. (a) Two‐way ANOVA with Tukey's post hoc test. (b) Non‐parametric independent‐samples Kruskal–Wallis test. Significance levels *p* < 0.05 are indicated by *, *p* < 0.01 by **, and *p* < 0.001 by ***. Purple dotted lines denote a significant difference between age groups when strains are combined (Table 5). Trabecular/cortical volume was significantly smaller in 18–20 and 40+ week mice compared to 8‐ to 11‐week‐old mice (*p* < 0.001). Trabecular thickness in CBA mice was significantly larger at 18–20 weeks versus 8‐ to11‐week‐old mice (*p* < 0.001).

Of the many parameters measured, time‐series profiling of trabecular volume relative to cortical volume was the only one to change significantly solely with age, while also being statistically unaffected by mouse strain (*p* = 0.369 for strain, *p* = 0.628 for strain/age interaction). For both strains, mouse trabecular volume relative to cortical volume declined steeply in co‐occurrence with skeletal maturation between 8–11 and 18–20 weeks of age (*p* < 0.001), and was then maintained in later life, a slight trend towards further decrease being non‐significant (*p* = 0.069). STR/Ort (OA) and CBA (healthy) mice appear indistinguishable from one another in this epiphyseal parameter throughout life (Figure 2a). Trabecular volume relative to cortical volume may therefore have utility in ageing a given epiphysis, but could not be used as an OA biomarker for these strains.

### 3.2 Cortical thickness was affected by mouse strain and age, but with limited biomarker utility, and trabecular thickness was statistically indistinguishable between strains

STR/Ort and CBA mice were also statistically indistinguishable from each other in terms of trabecular thickness at the three age timepoints investigated (Table 3), revealing this to be another poor biomarker in the context of our cross‐sectional study. However, CBA mice alone developed significantly thicker trabeculae between 8–11 and 18–20 weeks of age (*p* = 0.006), whilst STR/Ort mice only maintained their pre‐existing mean trabecular thickness between the same time points (Figure 2b, *p* = 0.429). This possible slight difference between the ageing trajectory of these strains means that it is possible for trabecular thickness to have some utility as a biomarker in future longitudinal OA studies, or cross‐sectional studies with an increased number of early‐life time points. Note that trabecular thickness was the only parameter that did not pass Levene's test; therefore, a Kruskal–Wallis test was used.

**FIGURE 3:**
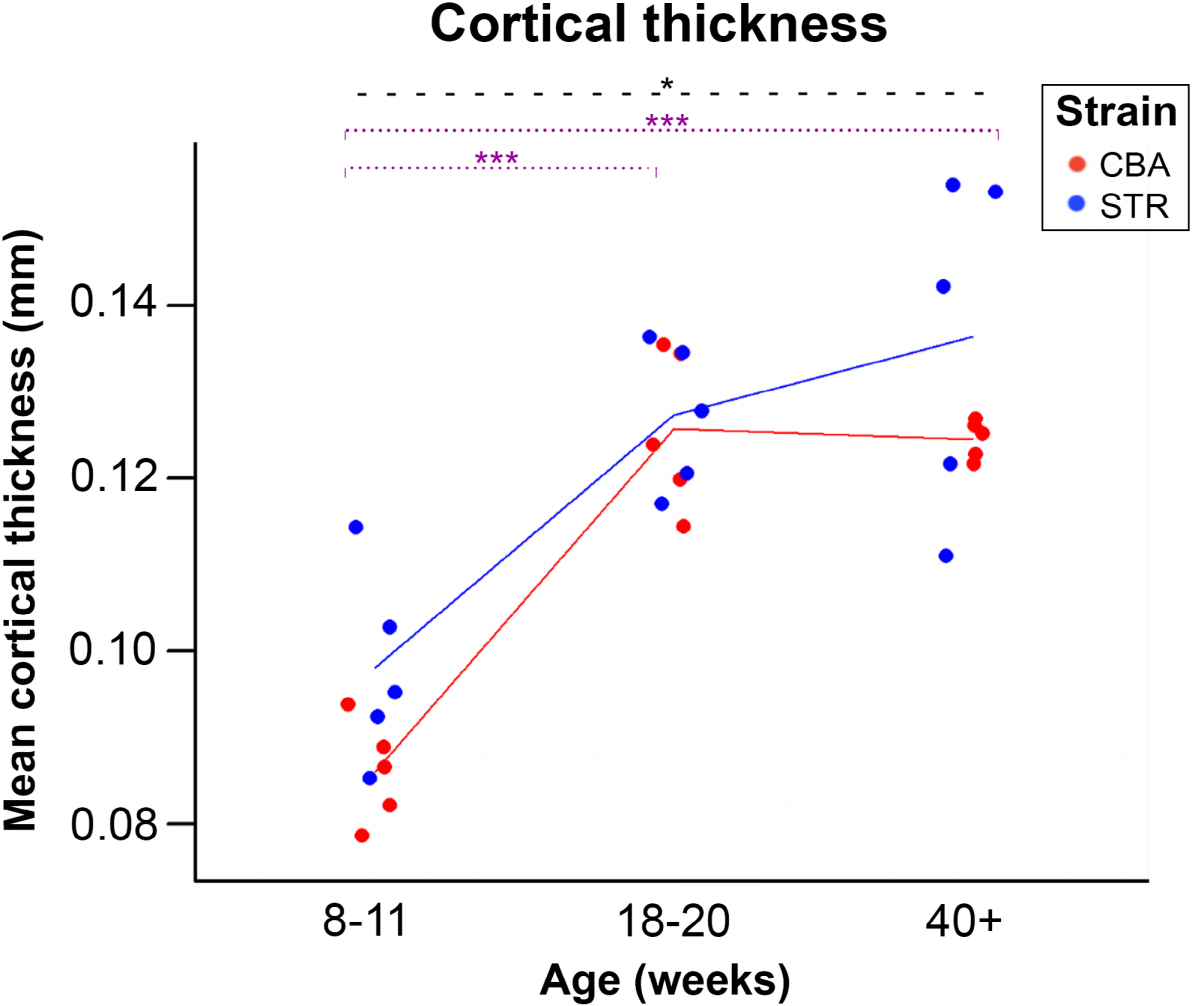
Cortical thickness is affected by mouse strain and age, but exhibits overlap between strains, showing poor promise as a biomarker. STR/Ort mice (blue) have significantly higher cortical thickness (*p* = 0.040) than CBA mice (red). Eight‐ to eleven‐week‐old mice have significantly lower cortical thickness than 18–20‐week‐old mice (*p* < 0.001) and than 40‐week‐old mice (*p* < 0.001). Significance levels *p* < 0.05 are indicated by *, *p* < 0.01 by ** and *p* < 0.001 by ***. Blue indicates STR/Ort mice (OA) and red indicates CBA mice (healthy). Purple dotted lines denote a significant difference between age groups when strains are combined (Table 5). Black dashed lines denote a significant difference between the two strains. Two‐way ANOVA with Tukey's post hoc test, significance level *p* < 0.05.

Cortical thickness was higher in STR/Ort (OA) than in CBA (healthy) mice (*p* = 0.040). Although this difference was consistent on average (Table 4) and detectable statistically, there are data value overlaps at all timepoints, with the two strains showing similar ageing trajectories (increasing from 8–11 to 18–20 weeks (*p* < 0.001), co‐occurring with the period of skeletal maturation) and no age/strain interaction (Figure 3). Cortical thickness may therefore prove to be a useful biomarker in large populations, and/or in combination with other joint anatomical features, but does not appear to have strong predictive or diagnostic power as a biomarker in and of itself given our results.

### 3.3 Cortical and trabecular volumes, and trabecular anisotropy exhibit conserved ageing‐related trajectories yet marked strain differences

**FIGURE 4:**
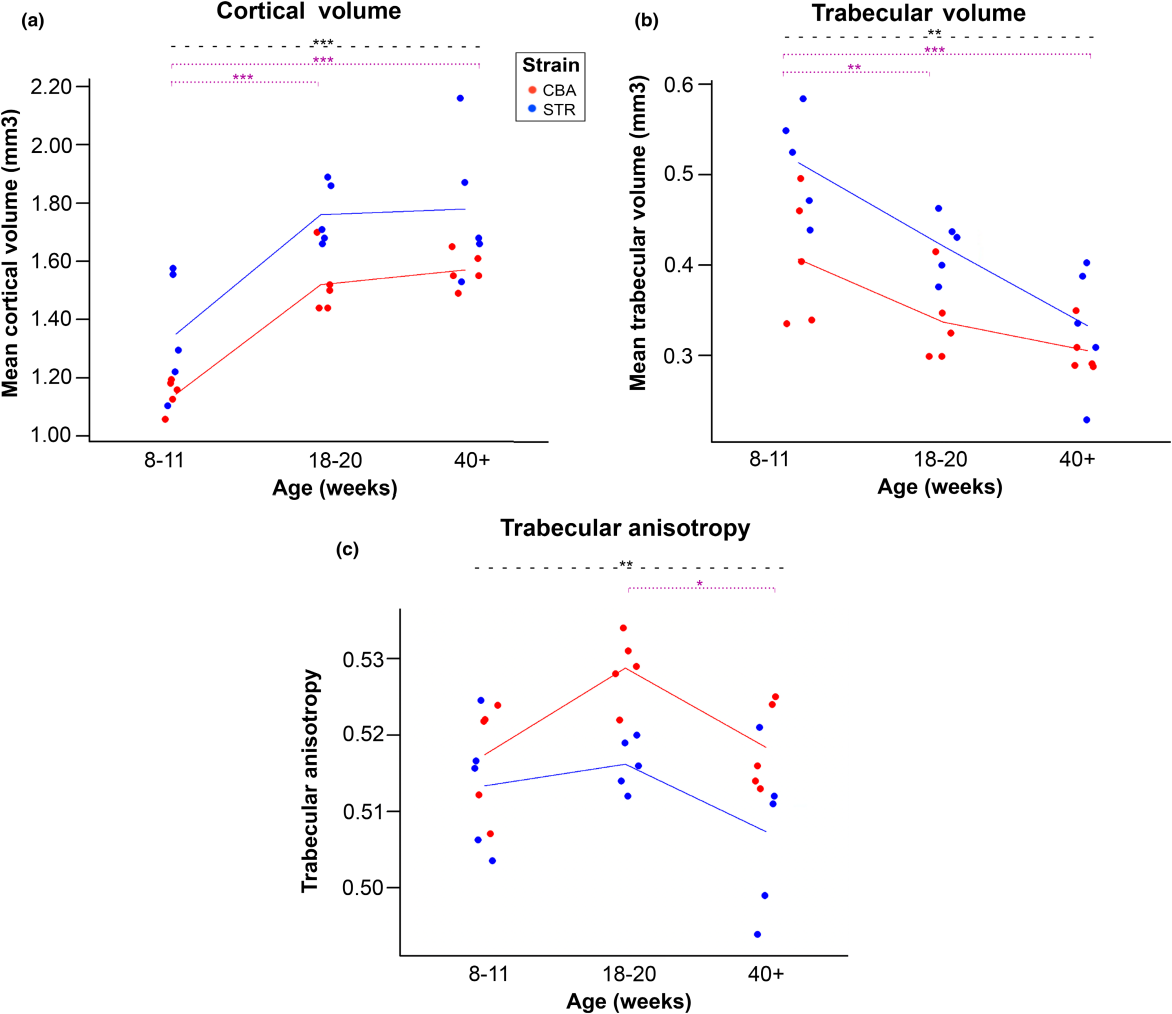
Cortical volume, trabecular volume and trabecular anisotropy differ between STR/Ort and CBA mice. These parameters also change with age, but there is no significant strain‐age interaction, meaning strains are differentiable throughout life and follow similar ageing trajectories. (a) STR/Ort mice have significantly greater cortical bone volume than CBA (*p* < 0.001), a feature that also increases significantly for both strains with age between 8–11 and 18–20 weeks (*p* = 0.001). (b) STR/Ort mice have significantly greater trabecular bone volume than CBA (*p* = 0.001), a feature which also decreases with age (*p* = 0.007 between 8–11 and 18–20 weeks, and *p* = 0.052 between 18–20 weeks and 40+ weeks). (c) STR/Ort mice have significantly lower trabecular anisotropy than CBA (*p* = 0.002), and trabecular anisotropy decreases significantly between 18–20 and 40 + weeks for both strains (*p* = 0.016). (a–c) Two‐way ANOVA with Tukey's post hoc test. * denotes *p* < 0.05, ** *p* < 0.01 and *** *p* < 0.001 by. STR/Ort (blue, OA) and CBA mice (red, healthy). Purple dotted lines denote a significant difference between age groups when strains are combined (Table 5). Black dashed lines denote a significant difference between the two strains.

STR/Ort mice differed significantly from CBA mice, with no detectable age‐strain interaction, in three probed epiphyseal parameters: cortical volume, trabecular volume, and trabecular degree of anisotropy (Table 2, Figure 4a–c). Cortical and trabecular bone volumes were both significantly greater in STR/Ort than CBA mice (*p*‐values ≤0.001), and anisotropy was significantly greater in CBA than in STR/Ort mice (*p* = 0.002). The lack of statistical age‐interaction effect indicates that these could be reliable biomarkers by which to differentiate the strains, and thus assess OA probability, throughout life, so long as mouse age is known.

Post hoc Tukey's tests (Table 5) confirmed that for both strains combined, 8‐ to 11‐week‐old mice have significantly less cortical bone volume than 18–20 (*p* < 0.001) and 40+‐week‐old (*p* < 0.001) mice, the latter two age groups having no significant difference in cortical bone volume from one another (*p* = 0.859, Figure 4a). Trabecular bone volume decreases with age for both strains (Figure 4b); 8‐ to 11‐week‐old mice have significantly greater trabecular volume than 18‐ to 20‐week‐old mice (*p* = 0.007), which in turn have near‐significantly greater trabecular volume than 40+‐week‐old mice (*p* = 0.052). Ageing, thus, is characterised in part by a reduction in the overall trabecular bone volume.

**TABLE 5:** *p*‐values identified from Tukey's post‐ hoc test applied to parameters where significant age differences (without age and strain interactions) were identified by ANOVA.

	Age 1 (weeks)	Comparator age (weeks)	*p*‐value
Cortical bone volume (mm^3^)	10	20	<0.001*
		40	<0.001*
	20	40	0.859
Trabecular bone volume (mm^3^)	10	20	0.007*
		40	<0.001*
	20	40	0.052
Trabecular volume relative to cortical volume	10	20	<0.001*
		40	<0.001*
	20	40	0.069
Trabecular degree of anisotropy	10	20	0.086
		40	0.722
	20	40	0.016*
Cortical thickness (mm)	10	20	<0.001*
		40	<0.001*
	20	40	0.687

* Indicates significance at the 0.05 level.

Trabecular anisotropy was significantly greater in CBA than STR/Ort mice (*p* = 0.002), evidently differentiating the two strains most clearly at 18–20 weeks (early OA‐onset, Figure 4c); at this time point, there is no overlap between groups. Post hoc Tukey's test (Table 5) showed that trabecular degree of anisotropy of the combined strains did not change significantly between 8–11 weeks and 18–20 weeks (*p* = 0.086, Table 5). Between 18–20 and 40+ weeks, however, a significant decline in anisotropy was observed in data from both strains (*p* = 0.016).

### 3.4 Marked strain differences in the ageing‐related trajectories of total epiphyseal volume, marrow space volume and trabecular ‘BV/TV’ expose potential imaging OA biomarkers

Mouse strain was also identified by ANOVA to have a significant effect in interaction with age for: total epiphyseal volume (*p* = 0.012), epiphyseal marrow space volume (MSV, *p* < 0.001) and trabecular BV/TV (*p* = 0.005). Interaction effects demonstrate that for these parameters, the two strains of mice differ from one another specifically with regard to the way in which these bony features change with age. Full pairwise simple main effects analyses using Fisher's LSD was performed to clarify these relationships.

**FIGURE 5:**
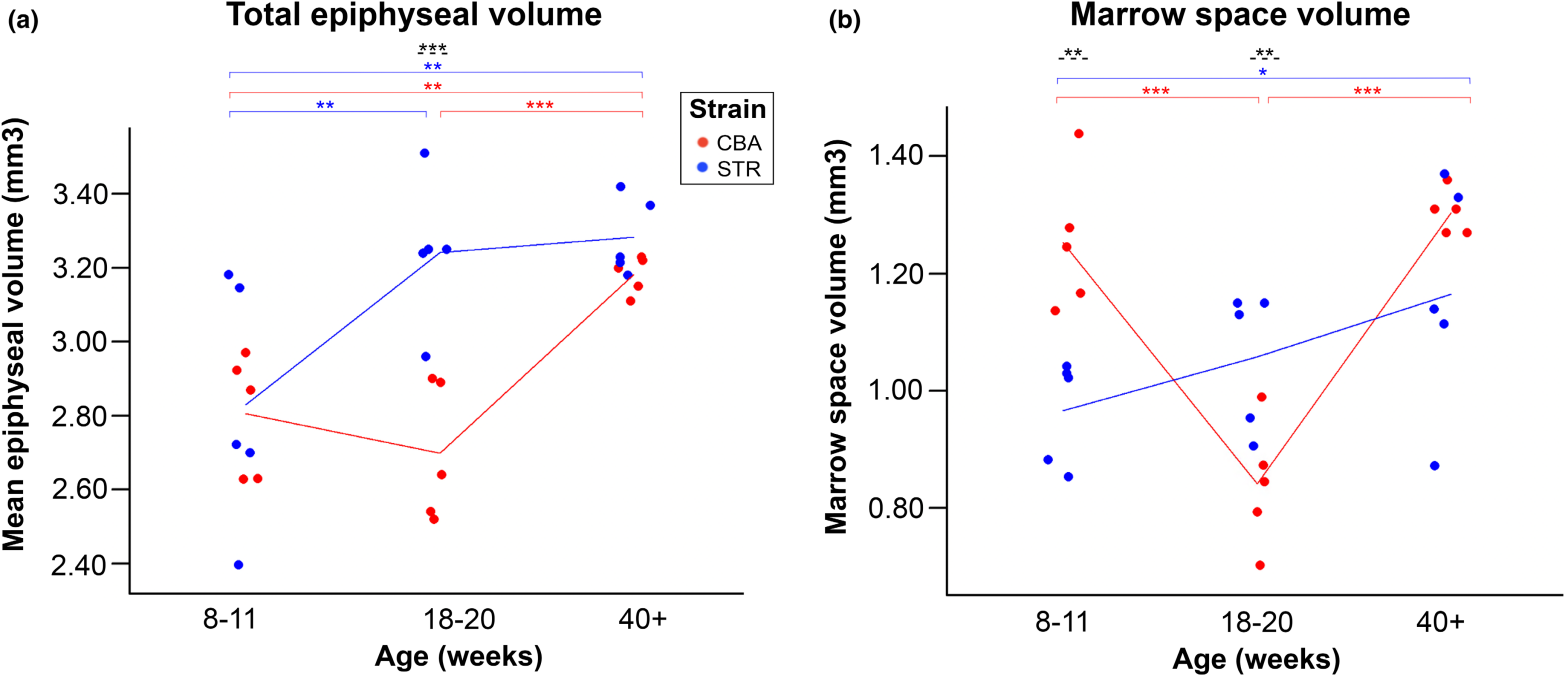
Total epiphyseal volume and marrows space volume (MSV) depend on strain, and strains show different ageing effects. (a) STR/Ort (OA, blue) and CBA (healthy, red) mice have no significant difference between epiphyseal volumes at 8–11 weeks (*p* = 0.841) and 40+ weeks (*p* = 0.416) of age, but their growth patterns are divergent, such that STR/Ort mice have a significantly greater epiphyseal volume than CBA at 18–20 weeks (*p* < 0.001), which represents early‐stage OA. Both mouse strains have significantly greater epiphyseal volumes at 40+ weeks than at 8–11 weeks of age (*p* < 0.006), and STR/Ort mice also have significantly greater total epiphyseal volume at 18–20 weeks versus 8–11 weeks (*p* = 0.002). (b) STR/Ort (OA, blue) mice reveal a significantly lower marrow space volume value than CBA mice at 8–11 weeks of age (*p* = 0.001), but this is reversed by 18–20 weeks, when STR/Ort MSV is significantly greater (*p* = 0.009) than that of their CBA (healthy, red) counterparts. There are no significant MSV differences detectable at 40+ weeks between the strains (*p* = 0.083). Eight‐ to eleven‐week‐old CBA mice have significantly higher marrow space volume than 18–20 week mice, and 18–20 week mice have significantly lower marrow space volume than 40+ week mice (both *p*‐values <0.001). For STR/Orts, only 40+ week mice are significantly different from 8‐ to 11‐week‐old mice (*p* = 0.016). Two‐way ANOVA with Tukey's post hoc test, significance levels *p* < 0.05 are indicated by *, *p* < 0.01 by ** and *p* < 0.001 by ***. Blue indicates STR/Ort mice (OA) and red indicates CBA mice (healthy). Black dashed lines denote a significant difference between the two strains.

STR/Ort had significantly greater total epiphyseal volume than CBA mice at 18–20 weeks (*p* < 0.001), but any differences between the two strains were non‐significant at both 8–11 weeks (*p* = 0.841) and 40+ weeks (*p* = 0.416) of age (Figure 5a). Tukey's post hoc analysis confirmed that for both strains combined, total epiphyseal volume increased significantly overall with growth between 8–11 and 40+ weeks (*p* < 0.001, Table 5).

**FIGURE 6:**
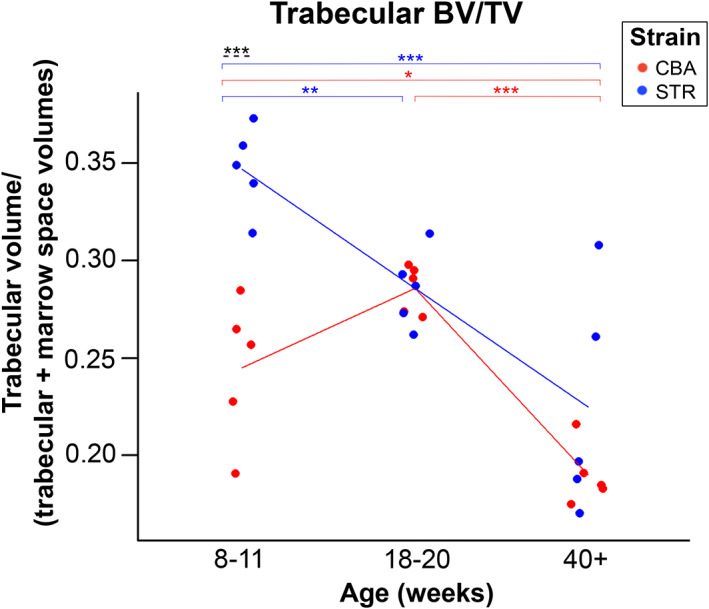
Trabecular BV/TV depends on strain, and strains show disparate ageing phenotypes. Blue indicates STR/Ort mice (OA) and red indicates CBA mice (healthy). There is no significant difference between the two strains at 18–20 weeks (*p* = 0.1.0) or 40+ weeks of age (0.092), but CBA have a significantly lower relative trabecular volume than STR/Ort at 8–11 weeks old (*p* < 0.001). Two‐way ANOVA with post hoc pairwise comparison test, significance levels *p* < 0.05 are indicated by *, *p* < 0.01 by ** and *p* < 0.001 by ***. Black dashed lines denote a significant difference between the two strains.

STR/Ort mice had significantly lower MSV than CBA mice at 8–11 weeks of age with no data overlap (*p* = 0.001), but this relationship was completely reversed by 18–20 weeks, by which point in the time series profiling the MSV of STR/Ort mice was significantly greater than those of CBA (*p* = 0.009, Figure 5(b)). No significant MSV difference was detectable between strains at 40+ weeks of age (*p* = 0.077). STR/Ort mice had significantly greater trabecular BV/TV than CBA mice at 8–11 weeks of age (*p* < 0.001), with no data overlap (Figure 6). Like MSV therefore, BV/TV is a highly promising biomarker of future OA development in these strains, suitable for use in early life in advance of any overt OA symptoms. This BV/TV difference between strains is entirely absent in later life at both 18–20 (*p* = 1.0) and 40+ weeks (*p* = 0.092) (Figure 6).

## 4 DISCUSSION

Our profiling of 3D epiphyseal architecture in CBA and STR/Ort mice with semi‐automated segmentation shows that: (i) while ageing affects all epiphyseal parameters, it is revealed as the exclusive factor in determining the ratio between trabecular and cortical bone volumes; (ii) despite exhibiting marked differences in magnitude between strains, the absolute volumes of the cortical and trabecular compartments, as well as anisotropy of the latter, display conserved age‐related behaviours in both strains, and finally (iii) strain differences in the ageing‐related trajectories of total epiphyseal volume, MSV and trabecular ‘BV/TV’ may identify imaging biomarkers of OA. These data yield new perspectives on the potential to identify pre‐symptomatic OA via imaging, and confirm gross anatomical differences between the OA‐prone tibial epiphyses of STR/Ort mice and healthy CBA counterparts. Some differences are present from 8–11 to 18–20 weeks of age, representing pre‐and very early‐stage OA respectively. In one study, almost all male STR/Ort mice showed cartilage lesions on the medial tibial plateau from 16 weeks and 100% had severe OA by 15 months (Javaheri et al., 2018); another demonstrated consistency in cartilage lesions occurring by 16 weeks, and lesion scores around 0 at 8–11 weeks (Poulet et al., 2012). Our data identify promising biomarkers for OA predisposition/progression at a macro‐anatomical scale that can readily be translated into human clinical practice.

This work adds to our understanding of the STR/Ort model of spontaneous, age‐related OA. Our work on CBA mice also yields baseline values characterising healthy ageing; this data is a useful reference for future studies in other skeletal pathologies by reveals insights into normal epiphyseal bone growth. Interestingly, we observed that standard deviations for parameters measured in STR/Ort mice were mostly greater than in CBA in older mice (Table 4); for example, in trabecular BV/TV, MSV and cortical volume at 40+ weeks (Figures 2a, 4a, 5b respectively). This aligns with results from Reznikov et al. that showed *broader scatter of values* in FEA‐simulated mechanical performance of OA and osteoporotic human femurs than healthy controls (Reznikov et al., 2020).

Our finding that trabecular: cortical volume ratio is determined by age independently of strain, with STR/Ort and CBA mice being statistically indistinguishable, as well as the lack of significant difference in trabecular thickness between strains at any age, suggests that they would make poor OA biomarkers. However, the significant influence of age suggests they may have clinical relevance in defining premature ageing phenotypes in other disorders. Conserved trabecular thickness and trabecular: cortical volume ratios may protect against other joint pathologies, such as Segond fracture local to the anterolateral ligament (Mullins et al., 2020). Interstrain similarities in multiple parameters of bone anatomy confirm the CBA as a suitable control for STR/Ort mice; their close degree of relatedness reducing the likelihood that the observed differences are wholly unrelated to OA. As STR/Ort mice are only one of potentially many specific age‐related OA endotypes, future studies may seek to confirm if these biomarkers differentiate age‐related from injury‐induced or other age‐related OA subtypes.

Studies have found greater cortical thickness with both increasing OA and OA versus healthy subjects, the latter of which was also true in our study. However, it must be noted that the high degree of overlap between groups is problematic for using cortical thickness as an OA biomarker. The relationship between trabecular thickness and OA is inconsistent in the literature. Auger et al. (2022) found no change in trabecular thickness with OA progression, and an increase in cortical thickness in human femoral necks. This agrees with our findings of increased cortical thickness at the early/middle age groups of STR/Ort mice (Table 3), no significant increase in trabecular thickness with age in STR/Or mice (in STR/Ort mice, ageing is linked to an increased OA phenotype), and no correlation between trabecular thickness and strain (where strain was a by‐proxy measure of OA). Note that all age groups in the Auger et al. (2022) study were skeletally mature, whereas in our study the first age group comprised juvenile mice. Finnila et al. (2017) also found increased SCBP thickness with OA severity and Grynpas et al. (1991) found cortical and trabecular thickness were higher in femoral heads of old OA patients (vs. old controls). Kamibayashi et al. (1995) found greater trabecular thickness in the tibia of OA patients^4^. Some of the differences between these studies could also be attributed to methodological differences: Auger et al. (2022) and Finnila et al. (2017) used 3D VOI analysis while Kamibayashi et al. (1995) and Grynpas et al. (1991) used histology, rather than 3D analyses of the whole subchondral region. Pishgar et al. (2021) used MRI‐based measures of trabecular thickness and trabecular BV/TV in medial subchondral bone and found that higher values of these metrics were correlated with an increased likelihood of medial tibial cartilage loss.

Significantly *lower* trabecular thickness in OA relative to non‐OA was found in cores of human patellae, femora, and tibiae (Hoechel et al., 2017; Patel et al., 2003; Reznikov et al., 2020). Stok et al. (2009) found mouse strain‐dependent architecture, with *thicker* trabeculae in both the femoral and tibial epiphysis of STR/Ort mice. They found cortical thickness to increase until 7 months, but that this only continued in advanced ages in CBA mice (Stok et al., 2009). This contrasts with our data showing increased cortical thickness from 8–11 to 18–20 weeks, which levelled off in both strains thereafter (Figure 3). These disparities may be explained by the regions analysed; Stok et al. included only proximal epiphysis cortical bone (extending just past the growth plate) whilst we included cortical bone of the entire epiphysis, up to but not including growth plate bridges (Herbst et al., 2021).

Some of our most promising findings are the statistical differences in cortical and trabecular bone volume, and trabecular anisotropy in STR/Ort and CBA mice; this is despite ageing profiles remaining similar in both strains. Future studies could investigate if tissue density differs between STR/Ort and CBA mice. Data from patients have shown bone density to be linked with OA severity, but whether this correlates with an increase or decrease in density depends on the study, methods used and type of density evaluated (Auger et al., 2022; Burr & Gallant, 2012; Grynpas et al., 1991; Hannan et al., 1993; Li & Aspden, 1997a, 1997b; Nevitt et al., 2010).

STR/Ort mice having greater cortical and trabecular bone volumes than CBA at all ages, including at 8–11 weeks (Figure 4a,b), indicates that these represent an OA risk factor attained prior to skeletal maturity. Stok et al. (2009) also found that STR/Ort mice had higher tibial subchondral cortical volume. Translationally, this suggests high cortical and/or trabecular bone volume may be a predictive OA biomarker in humans even in young adulthood, long before OA is typically diagnosed. However, it is worth emphasising that female STR/Ort mice have even higher average bone volume than male counterparts, yet no parallel increase in OA predisposition (Javaheri et al., 2018). Future research, translational or otherwise, should allow for such potential sexual dimorphism.

Anisotropy, a 3D measure of trabecular arrangement, was significantly lower in STR/Ort than CBA mice, with both strains exhibiting age‐related decline from ~18 to 40+ weeks (*p* = 0.016). Lower anisotropy means more three‐dimensionally symmetrical trabeculae. Significantly lower trabecular anisotropy values across all age groups in STR/Ort mice may suggest premature ageing in the trabecular arrangement. Indeed, the trabecular anisotropy values in STR/Ort mice even at 8–11 weeks are similar to the CBA anisotropy values at 40+ weeks (Figure 4c). Degree of epiphyseal trabecular anisotropy thus shows significant promise as an OA biomarker not only for its high sensitivity to differentiate STR/Ort from CBA mice at 20 weeks, but also in an evidenced ability to reveal a premature ageing phenotype; at least in these high bone mass animals, and therefore potentially in high bone mass humans. There is also scope to differentiate CBA and STR/Ort mice at other ages, perhaps with a larger sample size or in combination with other parameters; in our study, there is a lot of overlap between strains at weeks 8–11, and some overlap at weeks 40+. Reznikov et al. (2020) found that trabecular ‘blueprints’ (without considering thickness) differed in OA, and osteoporotic human femora differed from those of normal subjects, with a lower resistance to compressive stress, higher von Mises stress and more shear‐dominant elements (based on FE analysis of skeletonised VOIs). Discovery of the three exciting biomarkers (trabecular anisotropy, cortical volume and trabecular bone volume) reveals that epiphyseal analysis alone provides sufficient data, sensitive enough to differentiate OA from non‐OA knee joints at an early stage. This region is clearly identifiable and can be easily segmented even with little knowledge of anatomy. Tibial epiphyseal biomarkers are therefore promising and can be isolated from femoral, patellar and joint space characteristics, making meaningful analyses of clinical scans faster.

Our data disclose an apparent disparity between trabecular thickness and volume; thickness increased with age (in CBA mice between 8–11 and 18–20 weeks) and was not significantly different between the two strains, whereas volume decreased with age and was higher in STR/Ort than CBA. The greater trabecular volume in STR/Ort than CBA mice is therefore not a consequence of greater trabecular thickness. There are several other changes in trabecular architecture that could increase trabecular volume; these changes remain to be investigated in the context of our findings.

Our data touch upon the interaction between strain/age, which was confirmed as significant for total epiphyseal volume, MSV and trabecular BV/TV. This is intriguing as the effects of ageing are, crucially, different for STR/Ort and CBA mice. Although total epiphyseal volume was greater in STR/Ort than CBA, this was only transiently true at 18–20 weeks, indicating that this biomarker is only relevant during early OA. The total epiphyseal volume would therefore be a biomarker best used in combination with the aforementioned biomarkers of cortical and trabecular volume, and anisotropy which consistently differentiated STR/Ort OA, or OA‐prone, knees from healthy controls, but failed to specify any defined OA stage. Detecting a significantly higher total epiphyseal volume in addition to OA‐indications in these other biomarkers, then, could be highly useful as a biomarker specifically of early OA. Early‐OA biomarkers have perhaps the greatest potential for clinical impact, as cartilage lesions are impossible to detect radiographically and OA diagnoses conventionally rely on joint space narrowing (JSN) measurements which appear later. Early OA, prior to JSN, is also when targeted locomotory or pre‐emptive pharmaceutical treatments may prevent progression or even halt OA onset. Total epiphyseal volume is also easy to measure as it does not require the segmentation of trabecular and cortical bone necessary for other biomarkers.

Trabecular BV/TV differentiates pre‐OA STR/Ort vs age‐matched healthy CBA even earlier (8–11 weeks; Figure 6), but there is no inter‐strain difference at 18–20 and 40+ weeks (early‐ and late OA). This is likely, partially attributable to the relatively low MSV in 18‐ to 20‐week‐old CBA mice (Figure 5b), in combination with general decreases in trabecular volume through time in both strains and lower starting trabecular volume in CBA.

There is some disagreement in published studies on correlation between trabecular BV/TV (trabecular volume: epiphyseal interior) and OA. Renault et al. (2020) analysed tibial epiphyseal bone in varus and valgus OA patients, finding that elastic modulus and BV/TV on the overloaded side were linked with more severe OA (KL score). We found similar trends in STR/Ort, with lower trabecular BV/TV in older mice (Figure 6). On the other hand, Finnila et al. (2017) found that subchondral trabecular BV/TV in human tibial epiphyses *increased* with OA severity (OARSI grade). OA patients had higher trabecular BV/TV than healthy subjects in the superior (not inferior) part of the femoral head (Fazzalari & Parkinson, 1997), which also agrees with our findings that OA mice had higher trabecular BV/TV compared to controls (except 18–20 weeks). Rapagna et al. (2021) also found that in varus and valgus knee OA, the subchondral trabecular bone fraction was greater on the more loaded side (vs controls). The strength of this evidence is somewhat limited, however, as the effects of knee misalignment cannot be separated from the effects of OA, as the corresponding controls did not have either varus or valgus.

Cox et al. (2012) found that cartilage degeneration correlated with lower mean bone mineralisation and higher bone volume fraction in human OA tibial subchondral bone, and this trend was the strongest closest to the cartilage, suggesting a link between these phenomena. In 3D analysis of trabecular cores from OA/normal human femurs Reznikov et al. (2020) found that trabecular BV/TV was not different. Patel et al., 2003 found *lowe*r BV/TV with OA (vs controls) in cores of the proximal 6 mm of human tibiae. These discrepancies may rely on methodological differences; we find that age and strain affect trabecular BV/TV which may also explain these differing results.

Stok et al. (2009) found that BV/TV increased with age and showed strain: age interaction in the tibial trabeculae of both strains; BV/TV was similar in younger mice, and *lower* in STR/Ort than CBA mice at 3 months with only CBA mice exhibiting further increases with age. These studies also found BV/TV to be higher in femoral epiphysis in STR/Ort relative to CBA mice, with Stok et al. noting that the greater trabecular number in CBA could explain the higher BV/TV tibial epiphysis (Stok et al., 2009). We also found that trabecular BV/TV was both age and strain dependent, but revealed a differing age/strain relationship in the tibia, where STR/Ort had greater trabecular BV/TV at 8–11 weeks than CBA mice. In CBA mice, trabecular BV/TV increased between 8–11 and 18–20 weeks (nearly significant, *p* = 0.052), then decreased significantly between 18–20 and 40 + weeks (age range where BV/TV was increased in CBA in Stok et al., 2009), whereas STR/Ort BV/TV declined with age in our study (*p* ≤ 0.005 between all age groups). The differences between our study and Stok et al. (2009) could be affected by differences in colonies or housing environment, or possibly differences in image segmentation. More time‐series profiling is needed to resolve these different trends. At present, trabecular BV/TV is a promising biomarker for detecting specifically pre‐OA epiphyseal changes in the knee.

MSV can identify pre‐OA versus healthy controls without other biomarkers at 8–11 and 18–20 weeks, suggesting that this can sensitively monitor epiphyseal changes prior to OA onset. STR/Ort mice had significantly lower MSV than CBA at 8–11 weeks, which was completely reversed by 18–20 weeks, by which time MSV in STR/Ort was significantly greater than in CBA mice. Despite this statistical significance, at 18–20 weeks the two strains do have a single overlapping data point (Figure 5b), indicating that for early OA (18–20 weeks) MSV would also be more reliable if combined with other biomarkers for the purpose of individual diagnosis. MSV might be considered one of the most promising biomarkers of early OA, with a brief *window of opportunity* during which a pre‐OA, yet otherwise healthy joint state can be detected (Figure 5b). Future longitudinal studies could explore this phase to more precisely determine this MSV trajectory, whether there is a similarly reliable human equivalent to this observation, and whether this finding extends to other OA joints.

Total epiphyseal interior volume is not commonly reported; instead reporting trabecular BV/TV and parameters such as trabecular spacing, thickness and/or number that together could reflect changes in MSV. Thus, Stok et al. (2009) reported greater trabecular thickness in STR/Ort mice (linked to decreased MSV) but lower trabecular number (lined to increased MSV). The net effect of these trabecular changes on MSV is easiest to calculate by looking at overall MSV volume, which could also reveal changes in absolute growth of the epiphyseal interior (marrow space and trabecular bone) that might not be detected by the relative value of BV/TV. Investigating MSV changes with other parameters may indeed provide the most information about how the entire epiphysis changes between strains during ageing/OA. Nonetheless, MSV shows some promise as a biomarker of pre‐OA since it can distinguish between strains at 8–11 weeks (as does trabecular BV/TV). Future work could test whether early morphological differences between strains have mechanical implications for disease progression. We propose that future studies thus report total epiphyseal, marrow and trabecular and cortical volumes, as well as trabecular BV/TV and anisotropy; the latter providing relevant data for predicting stiffness (Maquer et al., 2015; Musy et al., 2017). Future studies need also to determine whether the small differences in trabecular anisotropy between OA and control mice have mechanical and functional consequences.

Epiphyseal changes with significant strain/age interaction, namely total epiphyseal volume, MSV and trabecular BV/TV demonstrate divergent ageing trajectories in STR/Ort and CBA mice and may therefore prove suitable biomarkers for monitoring drug treatment efficacy. Our data serve as a baseline for the typical age‐related effects in STR/Ort, and effectiveness could be assessed by determining if drugs restore the healthy ageing trajectories seen in CBA mice. A caveat to this study is the relatively small sample size that reduces the statistical power of our results. The lack of statistical significances in Levene's test (for homogeneity of variance) may also be due to low power. Additionally, differences between CBA and STR/Ort mice at young ages (e.g. MSV) could feasibly be strain differences unrelated to OA. Research in female STR/Ort (less OA predisposed) may help tease out these relationships, and further studies with larger datasets, or literature meta‐analyses, can build on our study in the future. It is worth noting here that in humans, females are more predisposed to knee OA, especially after the age of 55, when their sex hormones decrease (meta‐analysis by Srikanth et al., 2005). Exogenous oestrogen appears to have a protective effect on cartilage and bone in humans (Linn et al., 2012; Tanamas et al., 2011), but studies using ovariectomised female STR/Ort mice suggest that oestrogens have only a minor role in the relative protection from OA (Chambers et al., 1999). Therefore, future work is needed to explore the effects of endogenous sex hormones on radiographic OA and cartilage volume in more detail (Linn et al., 2012; Tanamas et al., 2011).

To transfer this data to clinical studies, an exploration of whether human OA patients and healthy subjects also show similar trends as 3D bone analysis could provide a consistent and unbiased metric to determine drug effects in OA. Future studies could also explore the mechanical links between changes in epiphyseal bone and cartilage pathology, for example, by using finite element modelling to investigate joint loading in healthy and OA knees (Reznikov et al., 2020). Finite element modelling could be used to assess how the epiphyseal structure of different strains and ages affects the mechanical environment and load distribution in these animals.

## 5 CONCLUSION

We used 3D analysis of entire epiphyses to characterise the effects of ageing, OA predisposition and progression on epiphyseal bone anatomy in mice. STR/Ort (OA) mice had higher cortical and trabecular volumes and lower anisotropy. OA and normal mice also differed in how total epiphyseal volume, MSV and trabecular BV/TV changed during growth (revealing characteristics of OA onset/progression). These parameters provide promising biomarkers for early diagnosis of OA, and for monitoring OA progression and efficacy of treatments. We also characterised the profile of normal skeletal changes in healthy mouse epiphyses that can serve as an important baseline for studies investigating the effects of various pathologies on skeletal structure and growth.

## AUTHORS’ CONTRIBUTION

E.C.H. designed the study, with contributions from A.A.F., L.A.E.E. and A.A.P; E.C.H. and L.A.E.E. wrote the manuscript and made the figures. L.A.E.E. conducted the statistical analysis. E.C.H., A.A.F. and L.A.E.E. segmented the scans and calculated the 3D metrics. B.J. provided scans for the STR/Ort and CBA mice. E.C.H., A.A.F., L.A.E.E., B.J. and A.A.P. all edited and revised the manuscript and approved submission of this manuscript.

## FUNDING INFORMATION

This work was funded by the OA Tech+ Network (grant EP/N027264/1) and the Anatomical Society (grant SSD 011018SEAL – v1‐011217) and a Medical Research Council ImagingBioPro Network Proof of Concept Award (MR/R025673/1).

## CONFLICT OF INTEREST STATEMENT

The authors declare that the research was conducted in the absence of any commercial or financial relationships that could be construed as a potential conflict of interest.

## Data Availability

The CT datasets generated and analysed for this study can be found in the Figshare repository.

## References

[joa13834-bib-0001] Auger, J.D. , Naik, A.J. , Murakami, A.M. , Gerstenfeld, L.C. & Morgan, E.F. (2022) Spatial assessment of femoral neck bone density and microstructure in hip osteoarthritis. Bone Reports, 16, 101155. 34984214 10.1016/j.bonr.2021.101155 PMC8693349

[joa13834-bib-0002] Burr, D.B. & Gallant, M.A. (2012) Bone remodelling in osteoarthritis. Nature Reviews Rheumatology, 8, 665–673. 22868925 10.1038/nrrheum.2012.130

[joa13834-bib-0003] Chambers, M.G. , Suri, N. , Cover, P. , Buckingham, J. , Visco, D. & Mason, R.M. (1999) Aggrecan degradation and osteoarthritis in STR/Ort mice occur independently of sex and sex hormone status. In: 45th annual meeting, Orthopedic Society. Anaheim, CA: Orthopedic Society, p. 447.

[joa13834-bib-0004] Conaghan, P.G. , Kloppenburg, M. , Schett, G. , Bijlsma, J.W. & Committee, E. O. A. H . (2014) Osteoarthritis research priorities: a report from a EULAR ad hoc expert committee. Annals of the Rheumatic Diseases, 73, 1442–1445. 24625626 10.1136/annrheumdis-2013-204660

[joa13834-bib-0005] Cox, L.G. , Van Donkelaar, C.C. , Van Rietbergen, B. , Emans, P.J. & Ito, K. (2012) Decreased bone tissue mineralisation can partly explain subchondral sclerosis observed in osteoarthritis. Bone, 50, 1152–1161. 22342798 10.1016/j.bone.2012.01.024

[joa13834-bib-0006] Das Neves Borges, P. , Vincent, T.L. & Marenzana, M. (2017) Automated assessment of bone changes in cross‐sectional micro‐CT studies of murine experimental osteoarthritis. PLoS One, 12(3), e0174294. 28334010 10.1371/journal.pone.0174294 PMC5363908

[joa13834-bib-0007] Domander, R. , Felder, A.A. & Doube, M. (2021) BoneJ2–refactoring established research software. Wellcome Open Research, 6, 37. 33954267 10.12688/wellcomeopenres.16619.1 PMC8063517

[joa13834-bib-0008] Doube, M. , Rimadoma , Rueden, C. , Felder, A.A. , Hiner, M. & Eglinger, J. (2021) bonej‐org/BoneJ2: styloid‐r11 (bonej‐7.0.11). Zenodo. Available from: 10.5281/zenodo.4635373

[joa13834-bib-0009] Fazzalari, N.L. & Parkinson, I.H. (1997) Fractal properties of subchondral cancellous bone in severe osteoarthritis of the hip. Journal of Bone and Mineral Research, 12, 632–640. 9101375 10.1359/jbmr.1997.12.4.632

[joa13834-bib-0010] Finnila, M.A.J. , Thevenot, J. , Aho, O.M. , Tiitu, V. , Rautiainen, J. , Kauppinen, S. et al. (2017) Association between subchondral bone structure and osteoarthritis histopathological grade. Journal of Orthopaedic Research, 35, 785–792. 27227565 10.1002/jor.23312 PMC5412847

[joa13834-bib-0011] Florea, C. , Malo, M.K. , Rautiainen, J. , Makela, J.T. , Fick, J.M. , Nieminen, M.T. et al. (2015) Alterations in subchondral bone plate, trabecular bone and articular cartilage properties of rabbit femoral condyles at 4 weeks after anterior cruciate ligament transection. Osteoarthritis and Cartilage, 23, 414–422. 25479166 10.1016/j.joca.2014.11.023

[joa13834-bib-0012] Ghouri, A. & Conaghan, P.G. (2021) Prospects for therapies in osteoarthritis. Calcified Tissue International, 109, 339–350. 32055890 10.1007/s00223-020-00672-9 PMC8403110

[joa13834-bib-0013] Grynpas, M.D. , Alpert, B. , Katz, I. , Lieberman, I. & Pritzker, K.P. (1991) Subchondral bone in osteoarthritis. Calcified Tissue International, 49, 20–26. 1893292 10.1007/BF02555898

[joa13834-bib-0014] Guermazi, A. , Roemer, F.W. , Felson, D.T. & Brandt, K.D. (2013) Motion for debate: osteoarthritis clinical trials have not identified efficacious therapies because traditional imaging outcome measures are inadequate. Arthritis and Rheumatism, 65, 2748–2758. 23861096 10.1002/art.38086

[joa13834-bib-0015] Hannan, M.T. , Anderson, J.J. , Zhang, Y. , Levy, D. & Felson, D.T. (1993) Bone mineral density and knee osteoarthritis in elderly men and women. The Framingham study. Arthritis & Rheumatism, 36, 1671–1680. 8250986 10.1002/art.1780361205

[joa13834-bib-0016] He, Y. , Gram, A. , Simonsen, O. , Petersen, K.K. , Karsdal, M.A. & Bay‐Jensen, A.C. (2014) AB0104 develop and evaluate a new modified Mankin score system with special attention to subchondral bone. Annals of the Rheumatic Diseases, 73, 838.23524886

[joa13834-bib-0017] Herbst, E.C. , Felder, A.A. , Evans, L.A.E. , Ajami, S. , Javaheri, B. & Pitsillides, A.A. (2021) A new straightforward method for semi‐automated segmentation of trabecular bone from cortical bone in diverse and challenging morphologies. Royal Society Open Science, 8, 210408. 34386254 10.1098/rsos.210408 PMC8334830

[joa13834-bib-0018] Hoechel, S. , Deyhle, H. , Toranelli, M. & Muller‐Gerbl, M. (2017) Osteoarthritis alters the patellar bones subchondral trabecular architecture. Journal of Orthopaedic Research, 35, 1982–1989. 27879001 10.1002/jor.23490

[joa13834-bib-0019] Hunter, D.J. , Nicolson, P.J.A. , Little, C.B. , Robbins, S.R. , Wang, X. & Bennell, K.L. (2019) Developing strategic priorities in osteoarthritis research: proceedings and recommendations arising from the 2017 Australian osteoarthritis summit. BMC Musculoskeletal Disorders, 20, 74. 30760253 10.1186/s12891-019-2455-x PMC6375218

[joa13834-bib-0020] Janvier, T. , Jennane, R. , Valery, A. , Harrar, K. , Delplanque, M. , Lelong, C. et al. (2017) Subchondral tibial bone texture analysis predicts knee osteoarthritis progression: data from the osteoarthritis initiative: Tibial bone texture & knee OA progression. Osteoarthritis and Cartilage, 25, 259–266. 27742531 10.1016/j.joca.2016.10.005

[joa13834-bib-0021] Javaheri, B. , Razi, H. , Piles, M. , De Souza, R. , Chang, Y.M. , Maric‐Mur, I. et al. (2018) Sexually dimorphic tibia shape is linked to natural osteoarthritis in STR/Ort mice. Osteoarthritis and Cartilage, 26, 807–817. 29604337 10.1016/j.joca.2018.03.008 PMC5987380

[joa13834-bib-0022] Kamibayashi, L. , Wyss, U.P. , Cooke, T.D. & Zee, B. (1995) Changes in mean trabecular orientation in the medial condyle of the proximal tibia in osteoarthritis. Calcified Tissue International, 57, 69–73. 7671169 10.1007/BF00299000

[joa13834-bib-0023] Kraus, V.B. , Collins, J.E. , Charles, H.C. , Pieper, C.F. , Whitley, L. , Losina, E. et al. (2018) Predictive Validity of Radiographic Trabecular Bone Texture in Knee Osteoarthritis. Arthritis & Rheumatology, 70, 80–87. 29024470 10.1002/art.40348 PMC5745253

[joa13834-bib-0024] Li, B. & Aspden, R.M. (1997a) Composition and mechanical properties of cancellous bone from the femoral head of patients with osteoporosis or osteoarthritis. Journal of Bone and Mineral Research, 12, 641–651. 9101376 10.1359/jbmr.1997.12.4.641

[joa13834-bib-0025] Li, B. & Aspden, R.M. (1997b) Mechanical and material properties of the subchondral bone plate from the femoral head of patients with osteoarthritis or osteoporosis. Annals of the Rheumatic Diseases, 56, 247–254. 9165997 10.1136/ard.56.4.247 PMC1752348

[joa13834-bib-0026] Linn, S. , Murtaugh, B. & Casey, E. (2012) Role of sex hormones in the development of osteoarthritis. PM&R, 4(5), S169–S173. 22632696 10.1016/j.pmrj.2012.01.013

[joa13834-bib-0027] Maquer, G. , Musy, S.N. , Wandel, J. , Gross, T. & Zysset, P.K. (2015) Bone volume fraction and fabric anisotropy are better determinants of trabecular bone stiffness than other morphological variables. Journal of Bone and Mineral Research, 30, 1000–1008. 25529534 10.1002/jbmr.2437

[joa13834-bib-0028] Mullins, W. , Jarvis, G.E. , Oluboyede, D. , Skingle, L. , Poole, K. , Turmezei, T. et al. (2020) The Segond fracture occurs at the site of lowest sub‐entheseal trabecular bone volume fraction on the tibial plateau. Journal of Anatomy, 237, 1040–1048. 32770847 10.1111/joa.13282 PMC7704226

[joa13834-bib-0029] Musy, S.N. , Maquer, G. , Panyasantisuk, J. , Wandel, J. & Zysset, P.K. (2017) Not only stiffness, but also yield strength of the trabecular structure determined by non‐linear microFE is best predicted by bone volume fraction and fabric tensor. Journal of the Mechanical Behavior of Biomedical Materials, 65, 808–813. 27788473 10.1016/j.jmbbm.2016.10.004

[joa13834-bib-0030] Nevitt, M.C. , Zhang, Y. , Javaid, M.K. , Neogi, T. , Curtis, J.R. , Niu, J. et al. (2010) High systemic bone mineral density increases the risk of incident knee OA and joint space narrowing, but not radiographic progression of existing knee OA: the MOST study. Annals of the Rheumatic Diseases, 69, 163–168. 19147619 10.1136/ard.2008.099531 PMC2935624

[joa13834-bib-0031] Patel, V. , Issever, A.S. , Burghardt, A. , Laib, A. , Ries, M. & Majumdar, S. (2003) MicroCT evaluation of normal and osteoarthritic bone structure in human knee specimens. Journal of Orthopaedic Research, 21, 6–13. 12507574 10.1016/S0736-0266(02)00093-1

[joa13834-bib-0032] Pishgar, F. , Guermazi, A. , Roemer, F.W. , Link, T.M. & Demehri, S. (2021) Conventional MRI‐based subchondral trabecular biomarkers as predictors of knee osteoarthritis progression: data from the osteoarthritis initiative. European Radiology, 31(6), 3564–3573. 33241511 10.1007/s00330-020-07512-2 PMC9583892

[joa13834-bib-0033] Poulet, B. , Ulici, V. , Stone, T.C. , Pead, M. , Gburcik, V. , Constantinou, E. et al. (2012) Time‐series transcriptional profiling yields new perspectives on susceptibility to murine osteoarthritis. Arthritis and Rheumatism, 64(10), 3256–3266. 22833266 10.1002/art.34572

[joa13834-bib-0034] Rapagna, S. , Roberts, B.C. , Solomon, L.B. , Reynolds, K.J. , Thewlis, D. & Perilli, E. (2021) Tibial cartilage, subchondral bone plate and trabecular bone microarchitecture in varus‐ and valgus‐osteoarthritis versus controls. Journal of Orthopaedic Research, 39, 1988–1999. 33241575 10.1002/jor.24914

[joa13834-bib-0035] Renault, J.B. , Carmona, M. , Tzioupis, C. , Ollivier, M. , Argenson, J.N. , Parratte, S. et al. (2020) Tibial subchondral trabecular bone micromechanical and microarchitectural properties are affected by alignment and osteoarthritis stage. Scientific Reports, 10, 3975. 32132556 10.1038/s41598-020-60464-x PMC7055326

[joa13834-bib-0036] Reznikov, N. , Alsheghri, A.A. , Piche, N. , Gendron, M. , Desrosiers, C. , Morozova, I. et al. (2020) Altered topological blueprint of trabecular bone associates with skeletal pathology in humans. Bone Reports, 12, 100264. 32420414 10.1016/j.bonr.2020.100264 PMC7218160

[joa13834-bib-0037] Roberts, B.C. , Thewlis, D. , Solomon, L.B. , Mercer, G. , Reynolds, K.J. & Perilli, E. (2017) Systematic mapping of the subchondral bone 3D microarchitecture in the human tibial plateau: variations with joint alignment. Journal of Orthopaedic Research, 35, 1927–1941. 27891668 10.1002/jor.23474

[joa13834-bib-0038] Rueden, C.T. , Schindelin, J. , Hiner, M.C. , Dezonia, B.E. , Walter, A.E. , Arena, E.T. et al. (2017) ImageJ2: ImageJ for the next generation of scientific image data. BMC Bioinformatics, 18, 529. 29187165 10.1186/s12859-017-1934-z PMC5708080

[joa13834-bib-0039] Seeman, E. (2008) Bone quality: the material and structural basis of bone strength. Journal of Bone and Mineral Metabolism, 26, 1–8. 18095057 10.1007/s00774-007-0793-5

[joa13834-bib-0040] Shane Anderson, A. & Loeser, R.F. (2010) Why is osteoarthritis an age‐related disease? Best Practice & Research. Clinical Rheumatology, 24, 15–26. 20129196 10.1016/j.berh.2009.08.006 PMC2818253

[joa13834-bib-0041] Srikanth, V.K. , Fryer, J.L. , Zhai, G. , Winzenberg, T.M. , Hosmer, D. & Jones, G. (2005) A meta‐analysis of sex differences prevalence, incidence and severity of osteoarthritis. Osteoarthritis and Cartilage, 13(9), 769–781. 15978850 10.1016/j.joca.2005.04.014

[joa13834-bib-0042] Staines, K.A. , Poulet, B. , Wentworth, D.N. & Pitsillides, A.A. (2017) The STR/Ort mouse model of spontaneous osteoarthritis – an update. Osteoarthritis and Cartilage, 25, 802–808. 27965138 10.1016/j.joca.2016.12.014 PMC5446355

[joa13834-bib-0043] Stok, K.S. , Pelled, G. , Zilberman, Y. , Kallai, I. , Goldhahn, J. , Gazit, D. et al. (2009) Revealing the interplay of bone and cartilage in osteoarthritis through multimodal imaging of murine joints. Bone, 45, 414–422. 19481620 10.1016/j.bone.2009.05.017

[joa13834-bib-0044] Tanamas, S.K. , Wijethilake, P. , Wluka, A.E. , Davies‐Tuck, M.L. , Urquhart, D.M. , Wang, Y. et al. (2011) Sex hormones and structural changes in osteoarthritis: a systematic review. Maturitas, 69(2), 141–156. 21481553 10.1016/j.maturitas.2011.03.019

